# Mortality Risk Among Patients With Influenza Illness Admitted to the ICU: A Systematic Review and Meta‐Analysis

**DOI:** 10.1111/irv.70073

**Published:** 2025-03-16

**Authors:** Pablo Suárez‐Sánchez, Jara Majuelos‐Melguizo, Marina Hinojosa‐Campos, Bélène Podmore, Iain A. Gillespie, Jennifer Han, Rosa Sloot, Dina Christensen

**Affiliations:** ^1^ OXON Epidemiology Madrid Spain; ^2^ GSK Stevenage UK; ^3^ GSK Upper Providence Pennsylvania USA; ^4^ GSK London UK

**Keywords:** influenza, intensive care unit, meta‐analysis, mortality, neuraminidase inhibitors

## Abstract

**Background:**

Despite vaccination programs and available treatments, seasonal influenza carries a large mortality burden, especially in intensive care unit (ICU) settings. Understanding the influenza mortality burden in ICU settings can inform treatment planning and resource allocation. Nonetheless, surveillance data on mortality in ICU‐admitted patients are scarce and estimates vary greatly. This systematic literature review (SLR) and meta‐analysis investigated all‐cause mortality risk among ICU‐admitted patients with influenza in Europe.

**Methods:**

We included observational studies conducted in Europe that reported mortality among patients ≥ 6 months of age with influenza admitted to the ICU. Studies published between January‐2009 and December‐2019 were included. Quality was assessed using a modified Newcastle‐Ottawa scale. Pooled all‐cause mortality risk was calculated as a proportion using a random‐effects model with an inverse variance method. A sensitivity analysis was also conducted, including only studies identified as having low risk of bias.

**Results:**

Thirty‐seven studies, reporting on 13,616 patients, were included. All‐cause mortality ranged from 0% to 67%. The overall pooled mortality risk estimate was 0.24 (95% CI: 0.20, 0.27). Study heterogeneity was high (Cochran's Q test *p* < 0.01, I^2^ = 93%). The sensitivity analysis using only studies identified as having low risk of bias produced a pooled mortality risk of 0.25 (95%CI: 0.21, 0.29).

**Conclusions:**

These results indicate that approximately a quarter of patients with influenza admitted to the ICU die, reinforcing the need for effective vaccination programs and treatment optimization.

## 1 Introduction

Influenza is an infectious respiratory disease primarily caused by the influenza A and B viruses, affecting an estimated 5%–10% of adults and 20%–30% of children worldwide [1], and 10%–30% of the population in Europe [2], annually. For most patients, influenza is self‐limiting; however, certain factors, such as the extremes of age, residing in a long‐term care facility or nursing home, pregnancy, obesity, immunosuppression, diabetes mellitus and chronic cardiopulmonary, renal, hepatic, or neurologic illnesses put individuals at higher risk for developing complicated influenza [3, 4]. Complicated influenza is defined as illness in which hospitalization is required and/or with symptoms and signs of serious illnesses such as bronchitis, pneumonia, acute respiratory distress, myocarditis, encephalitis, and/or exacerbation of an underlying condition [3, 4].

Vaccination is the most effective way to prevent influenza illness, complications, and deaths and is estimated to reduce overall mortality by 39%–75% [1]. However, influenza vaccine uptake is suboptimal in many high‐risk groups [5]. In patients infected with influenza at high risk for developing complicated influenza, neuraminidase inhibitors (NAIs) are recommended as first‐line treatment: [6] oseltamivir phosphate, zanamivir, and baloxavir marboxil are authorized for use in the European Union (EU) and oseltamivir phosphate and baloxavir marboxil in the United States (US) for patients at risk of and/or with complicated influenza [7, 8]. Additionally, peramivir is approved in the US for the treatment of patients with uncomplicated influenza [8]. However, despite these available treatments seasonal influenza results in approximately 3–5 million severe infections and an estimated 290,000–650,000 deaths worldwide annually, with 28,000–73,000 deaths reported in Europe alone [9].

Patients with influenza who require hospitalization are at the highest risk for mortality. Specifically, patients who require treatment in the intensive care unit (ICU) are a subpopulation with a particularly high mortality burden. Additionally, ICU beds are costly, requiring specialized equipment and staffing [10]; furthermore, ICU beds are in high demand and units can be overwhelmed during pandemic years or when outbreaks of respiratory disease are severe – protocols to prevent the spread of droplet infections are particularly burdensome. ICU mortality estimates for influenza can provide hospitals with accurate information for guiding and evaluating the effectiveness of treatment strategies in this critically ill population, as well as help inform optimal use of healthcare resources [11].

European countries have been conducting influenza surveillance for years [12], monitoring the geographic spread, intensity of transmission, genetic evolution of the influenza virus, and the impact and severity of the disease (hospitalized cases, ICU cases and deaths) [13]. However, granular surveillance data on mortality among cases requiring admission to the ICU are scarce and difficulties in estimating disease burden persist, hence estimates within ICU‐admitted populations vary widely [14].

Therefore, this systematic literature review and meta‐analysis estimated the overall all‐cause mortality risk among patients with influenza admitted to ICUs in Europe, and thereby aimed to provide a more current and comprehensive picture of the disease landscape.

## 2 Methods

This systematic literature review and meta‐analysis was conducted in accordance to the Preferred Reporting Items for Systematic Reviews and Meta‐Analyses (PRISMA) guidelines [15].

### 2.1 Eligibility Criteria and Screening

A literature search on mortality risk among patients with influenza admitted to ICUs was conducted in the PubMed database on 22 April 2022. Observational studies published between 1 January 2009 and 31 December 2019 that reported on mortality outcomes in patients ≥ 6 months of age with influenza and admitted to the ICU were eligible for inclusion. The starting point of 1 January 2009 was selected to include the onset of the H1N1 influenza pandemic (April), during which the influenza A(H1N1)pdm09 strain displaced the previously circulating seasonal H1N1 strains and has since continued to circulate as the predominant H1N1 strain alongside other seasonal influenza viruses [16, 17, 18]. The search string used is shown in Table S1. Additionally, studies were required to be published in English, French, German, or Spanish languages, and conducted in countries within the EU‐27, the European Free Trade Association (EFTA) or in the United Kingdom (UK).

The reference lists of selected articles were checked to identify potential additional studies missed through the search. Reviews and meta‐analyses were excluded; however, their bibliographies were checked for relevant original studies. Any additional studies identified through these reference searches were included for further screening and selection.

Each screening step was performed by two independent reviewers and in all cases, disagreements were resolved through reaching consensus with a third reviewer. Screening, in which publications were assessed for eligibility based on the pre‐defined criteria, was conducted first by titles and abstracts, after duplicates were removed. The full text of articles that could not be excluded via title and abstract assessment was retrieved and assessed for eligibility.

### 2.2 Data Extraction and Outcome Measure

Data were extracted independently by two reviewers via a standardized form used for the assessment of study quality and evidence synthesis. Any discrepancies were resolved through discussion or via a third reviewer if necessary. Extracted information included study ID (first author and year of publication), study title, country/countries, study design, study period, patient source, study population characteristics, number of deaths, number of ICU‐admitted individuals with influenza, percentage female, age, percentage of cases with ≥ 1 risk factor, percentage of cases with laboratory‐confirmed influenza, percentages of cases with influenza A(H1N1), percentage of cases treated with NAI, percentage of vaccinated cases, and risk of bias.

### 2.3 Quality Assessment

The quality of the eligible studies was assessed using a Newcastle‐Ottawa scale [19] modified to better suit the objective of this study: as proportions were being estimated, criteria focusing on comparator groups (e.g., assessing the selection of the non‐exposed cohort and cohort comparability) were removed. Additionally, modifications were made to the exposure ascertainment (confirmed influenza, diagnosed by a physician, or no description) and outcome assessment (death occurring in the ICU clearly defined in ICU, or unclear) to better reflect the specific exposure and outcome of interest.

Each item could receive one star, except for the ascertainment of exposure, where the possibility of an additional star was included for studies that reported confirmed influenza. Based on the results of the quality assessment, studies were classified according to the risk of bias: low (selection domain: 3‐4 stars; AND outcome domain: 2‐3 stars), moderate (selection domain: 1‐2 stars; AND outcome domain: 2‐3 stars), and high (selection domain: 0 stars; OR outcome domain: 1‐2 stars). Each study was assessed independently by two reviewers. Disagreement was resolved by discussion and participation of a third reviewer when necessary. The full modified assessment can be found in Table S2.

### 2.4 Data Analysis

All‐cause mortality risk among patients with influenza who had been admitted to the ICU, which will be referred to as “all‐cause ICU mortality risk” from here on, was defined as [ICU‐admitted individuals with influenza who died from any cause] divided by [total number of ICU‐admitted individuals with influenza] and expressed as a proportion.

Heterogeneity between studies was evaluated using Cochran's Q test and I^2^ statistic, and the pooled estimate was derived using a random‐effects model, both in the main meta‐analysis and the sensitivity analysis, to account for potential variability across studies. Between‐study variance was estimated using the Der Simonian and Laird method [20]. An inverse variance method with a continuity correction of 0.5 in studies with zero cell frequencies and untransformed proportions was used. A sensitivity analysis incorporating only studies evaluated as having a low risk of bias was conducted to assess the impact of the risk of bias on the pooled estimate from the main analysis.

All analyses were conducted with R version 4.2.0 using the “metafor” (Version 3.4‐0) and “meta” (Version 5.2‐0) packages.

## 3 Results

### 3.1 Study Characteristics and Heterogeneity

**FIGURE 1:**
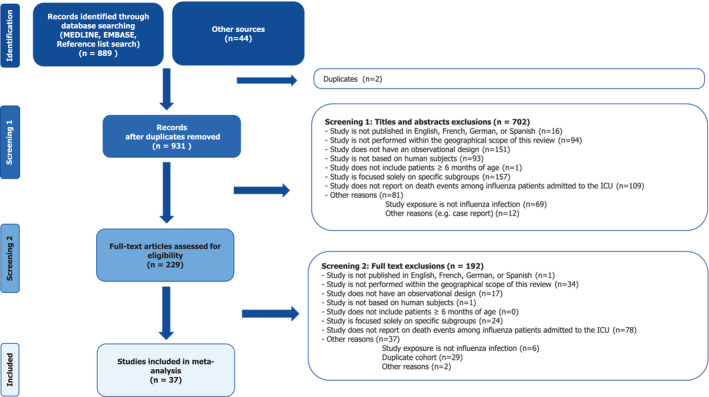
PRISMA flowchart for study selection and inclusion.

The initial literature search yielded 933 citations, of which 889 were found through PubMed, and 44 were found through other sources. Removal of 2 duplicate articles resulted in 931 citations for initial screening. Of these, 229 citations underwent full article assessment, resulting in 37 studies [21, 22, 23, 24, 25, 26, 27, 28, 29, 30, 31, 32, 33, 34, 35, 36, 37, 38, 39, 40, 41, 42, 43, 44, 45, 46, 47, 48, 49, 50, 51, 52, 53, 54, 55, 56, 57] reporting on 13,616 patients meeting the eligibility criteria for inclusion in the meta‐analysis (Figure 1).

Of the 37 studies, 13 reported a retrospective cohort design and 24 were prospective (Table 1). The majority of studies came from Spain (*n* = 8), Germany (*n* = 4), Belgium (*n* = 3), Greece (*n* = 3), Italy (*n* = 3), and the UK (*n* = 3). Two studies came from Romania (one of which was a multi‐country study where Romania was the only country of interest), and there was one study for each of the following countries: Austria, Denmark, Finland, France, Ireland, Lithuania, Netherlands, Norway, Slovakia, Sweden, and Switzerland. Through the quality assessment, 25 were classified as having a low risk of bias, with 9 classified as moderate and 3 as high. The most common drivers of bias were: reliance on clinical diagnosis rather than laboratory confirmation, inadequate information on follow‐up, and unclear information on the occurrence of death (e.g., not clearly stated if recorded from ICU or hospital discharge records).

**TABLE 1:** Characteristics of selected studies.

Study ID	Country	Study design	Study period	Patient source	Number of influenza cases (N)	Female (%)	Age (years)	Risk Of bias	All‐cause ICU mortality risk (%)
**Adenji 2011**	United Kingdom	Retrospective	Jul‐2009 to Feb‐2010	Hospital; Single centre	19	53	53 (median)	Low	15.8
**Adlhoch 2012**	Germany	Prospective (surveillance)	30‐Nov‐2009 to 31‐Mar‐2010	Hospital; National; Surveillance	59	NA	NA	Low	27.1
**Akers 2017**	Switzerland	Retrospective	Seasons 2013/2014 and 2014/2015	Hospital; Single centre	41	NA	NA	Low	17.1
**Athanasiou 2011**	Greece	Prospective (surveillance)	4‐Oct‐2010 to 22‐May‐2011	ICU; National; Surveillance	364	44	52 (median)	Low	39.6
**Ausselet 2014**	Belgium	Prospective	1‐Aug‐2009 to 31‐Dec‐2009	Hospital; Single centre	5	NA	NA	High	0.0
**Bassetti 2010**	Italy	Retrospective	1‐Jul‐2009 to 30‐Nov‐2009	Hospital; Multi‐centre	10	NA	43.5 (median)	High	20.0
**Bauernfeind 2013**	Germany	Retrospective	Seasons 2007/8 to 2010/11	Hospital; Single centre	51	NA	NA	Low	23.5
**Bertolini 2011**	Italy	Prospective	Oct‐2009 to Apr‐2010	ICU; Multi‐centre; Registry	315	43	43 (mean)	Low	17.1
**Beumer 2018**	Netherlands	Retrospective	1‐Oct‐2015 to 1‐Apr‐2016	Hospital; Multi‐centre	45	53	53.02 (mean)	Low	37.8
**Bonmarin 2015**	France	Prospective (surveillance)	Seasons 2009/2010 to 2012/2013	ICU; National; Surveillance	2676	45	46.68 (mean)	Low	21.3
**Brandsaeter 2011**	Norway	Prospective	15‐Jul‐2009 to 30‐Nov‐2009	Hospital; Single centre	17	NA	NA	Low	23.5
**Brink 2012**	Sweden	Retrospective	Aug‐2009 to Feb‐2010	ICU; Multi‐centre; Registry	126	44	44 (median)	Low	11.1
**Cardeñosa 2011**	Spain	Prospective (surveillance)	24‐Apr‐2009 to 20‐Jan‐2010	Hospital; Regional; Surveillance	284	NA	NA	Low	13.7
**Cherifi 2011**	Belgium	Retrospective	1‐Jun‐2009 to 30‐Nov‐2009	Hospital; Single centre	11	NA	NA	Low	9.1
**Chippirraz 2011**	Spain	Prospective	Jun‐2009 to Jan‐2010	Hospital; Single centre	10	NA	NA	Moderate	20.0
**Domínguez 2018**	Spain	Prospective (surveillance)	Seasons 2010–2011 to 2015–2016	Hospital; Regional; Surveillance	595	38	NA	Low	21.5
**Drăgănescu 2019**	Romania	Prospective (surveillance)	11‐Dec‐2017 to 30‐Apr‐2018	Hospital; Single centre; Surveillance	11	NA	NA	Moderate	9.1
**Gubbels 2012**	Denmark	Prospective (surveillance)	Seasons 2009–2010 and 2010–2011	ICU; National; Surveillance	201	44	49.89 (mean)	Low	27.4
**Heyd 2017**	Germany	Retrospective	25‐Dec‐2014 to 3‐May‐2015	Hospital; Multi‐centre	149	NA	NA	Low	29.5
**Hlavinkova 2015**	Slovakia	Prospective (surveillance)	28‐May‐2009 to 30‐Dec‐2009	Hospital; National; Surveillance	43	NA	NA	Low	67.4
**Lehners 2013**	Germany	Retrospective	May‐2009 to Apr‐2011	Hospital; Single centre	49	38	47.9 (mean)	Low	26.5
**Linko 2011**	Finland	Prospective	11‐Oct‐2009 to 31‐Dec‐2009	ICU; Multi‐centre; National	132	36	47.82 (median)	Low	7.6
**Lytras 2019**	Greece	Prospective (surveillance)	Seasons 2010–2011 to 2018–2019	ICU; National; Surveillance	2325	NA	NA	Low	39.7
**Martínez Ochoa 2010**	Spain	Prospective (surveillance)	Week 28–2009 to 3–2010	Hospital; Regional; Surveillance	5	40	41.8 (mean)	Moderate	40.0
**Martin‐Loeches 2016**	Spain	Prospective	Seasons 2009, 2010, 2014, 2015	ICU; Multi‐centre; Registry	2684	41	51.6 (mean)	Low	22.1
**Meerhoff 2015**	Multicountry_Romania extracted	Prospective (surveillance)	2009 to 2012	Hospital; Multi‐country; Surveillance	96	50	33 (median)	Low	31.3
**Mickienė 2011**	Lithuania	Retrospective	1‐Nov‐2009 to 15‐Mar‐2010	Hospital; Multi‐centre	9	66	40.1 (mean)	Moderate	66.7
**Nicolay 2010**	Ireland	Prospective	15‐Jul‐2009 to 30‐May‐2010	ICU; Multi‐centre; National	76	49	43 (median)	Low	18.4
**Pérez‐Carrasco 2015**	Spain	Prospective	2011/12, 2012/13 and 2013/14 seasons	ICU; Single centre	41	44	54 (median)	High	14.6
**Poeppl 2011**	Austria	Prospective	20‐Sep‐2009 to 02‐Feb‐2010	Hospital; Multi‐centre	47	43	46.9 (mean)	Low	27.7
**Rizzo 2016**	Italy	Prospective (surveillance)	Season 2010/2011 to 2014/15	Hospital; National; Surveillance	102	43	63 (median)	Moderate	23.5
**Rovina 2014**	Greece	Retrospective	Apr‐2009 to Dec‐2010	Hospital; Single centre	12	50	52 (mean)	Moderate	8.3
**Rowan 2010**	United Kingdom	Prospective	03‐Sep‐2009 to 31‐Jan‐2010	ICU; Multi‐centre; National	1651	50	44.05 (mean)	Moderate	19.4
**Santa‐Olalla Peralta 2010**	Spain	Prospective (surveillance)	24‐Apr‐2009 to 31‐Jan‐2010	ICU; National; Surveillance	1231	47	40 (median)	Low	22.0
**Scriven 2009**	United Kingdom	Retrospective	01‐Jun‐2009 to 21‐Jul‐2009	Hospital; Single centre	7	80 (2 NA)	38 (mean) (2 NA)	Moderate	14.3
**Van Ierssel 2014**	Belgium	Retrospective	2009 and winter 2010–2011	ICU; Single centre	16	37	42.8 (mean)	Moderate	43.8
**Viasus 2011**	Spain	Prospective	12‐Jun‐2009 to 10‐Nov‐2009 and 01‐Dec‐2009 to 31‐Mar‐2011	Hospital; Multi‐centre	101	NA	NA	Low	28.7

Abbreviations: ICU, intensive care unit; NA, not applicable.

The population size (ICU‐admitted individuals with influenza) ranged from 5 to 2684 individuals. The median percentage of female patients ranged from 36% to 80%, while the median age ranged from 33 to 63 years. In total, 32% (12/37) of studies excluded children below 16 (2 studies) or 18 years of age (10 studies), and 65% (24/37) of studies did not focus exclusively on patients in the ICU setting (only data on the ICU‐admitted patients were included in the meta‐analysis).

Additional study characteristics can be found in Tables S3 and S4. Briefly, 28 studies provided information on the percent of cases of the H1N1 strain, ranging from 0% to 100%. Additionally, 68% (25/37) of studies examined exclusively the season that coincided with the 2009 influenza pandemic (2009–2010) or the following season (2010–2011) which were dominated by influenza A(H1N1)pdm09 strain. The 2009–2010 season was exclusively covered in 51% (19/37) of studies. The proportion of patients with at least one risk factor was reported in 25 studies and ranged from 24% to 100%. The reported risk factors varied widely across the included studies, but commonly included immunosuppression, metabolic diseases, cardiovascular disease, neurocognitive disease, and other chronic diseases. The majority of studies (86% [32/37]) included 100% of patients with laboratory‐confirmed influenza, and 22% (8/37) studies reported vaccination status (ranging from 0% to 19% of patients being vaccinated). Treatment with NAIs was reported in 35% (13/37) of studies, with the percentage of patients receiving NAI treatment ranging from 57% to 100%.

### 3.2 Pooled Mortality Risk Among Patients With Influenza Admitted to the ICU

**FIGURE 2:**
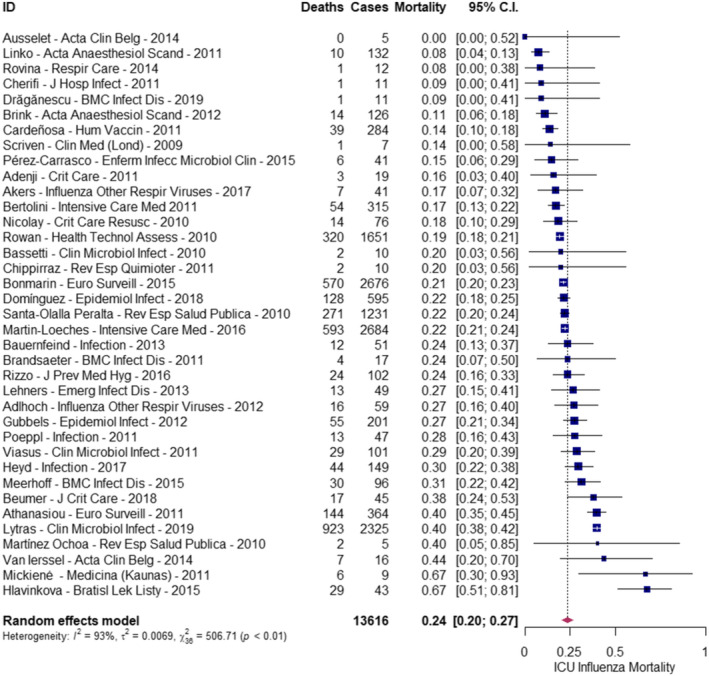
Pooled mortality risk among patients with influenza admitted to the ICU.

All‐cause ICU mortality risk ranged from 0% to 67% across the studies. Of the 13,616 patients admitted to the ICU with influenza, 3405 (25%) died. The overall pooled estimate of mortality risk was 0.24 (95% CI: 0.20, 0.27) (Figure 2). Heterogeneity was high (Cochran's Q test *p* < 0.01; I^2^: 93%).

### 3.3 Sensitivity Analysis

**FIGURE 3:**
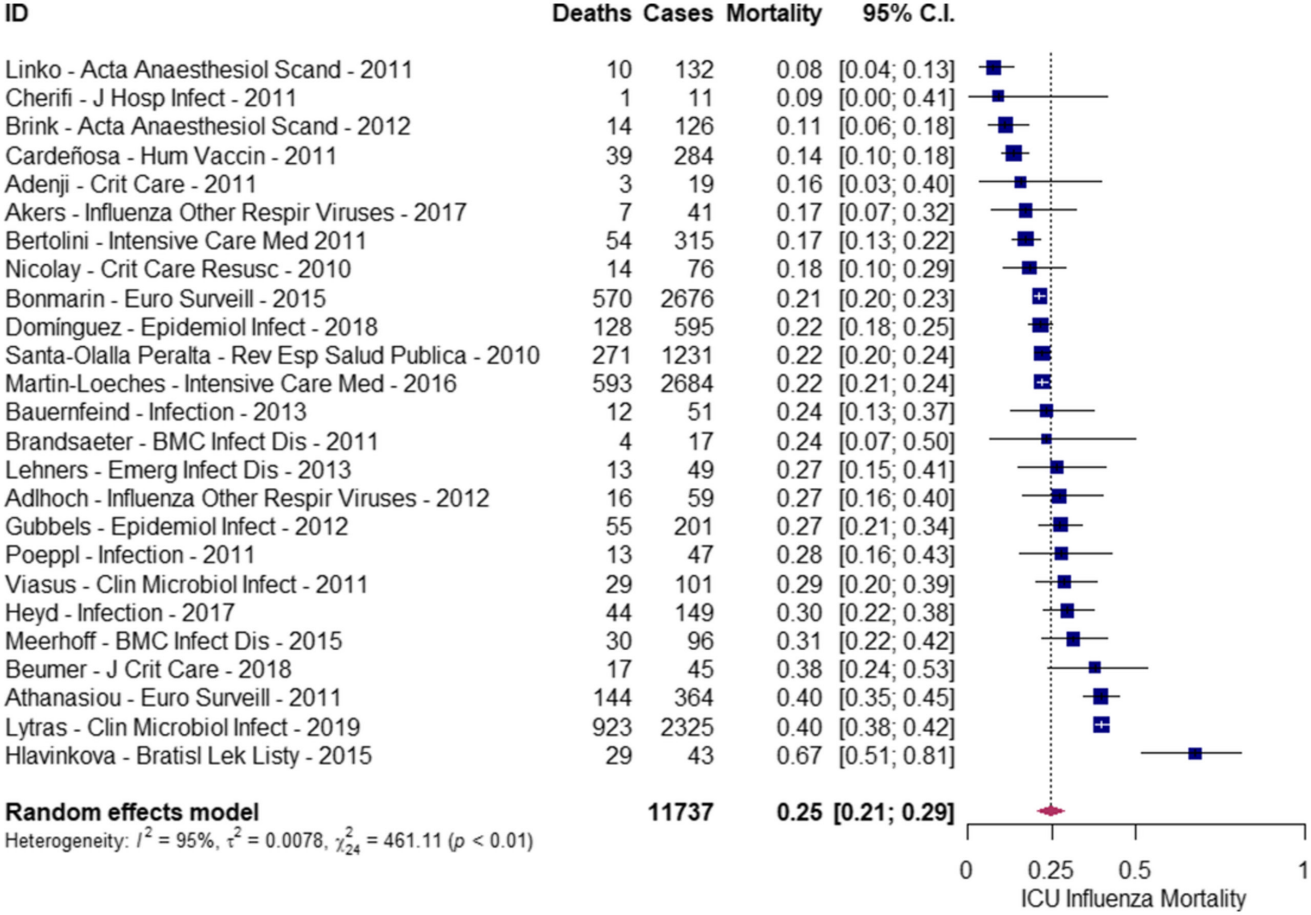
Pooled mortality risk among patients with influenza admitted to the ICU in studies with low risk of bias.

When considering only studies evaluated as having a low risk of bias, the reported all‐cause ICU mortality risk ranged from 8% to 67%. The pooled mortality risk estimate was 0.25 (95%CI: 0.21, 0.29) (Figure 3). Heterogeneity was high (Cochran's Q test, *p* < 0.01; I^2^: 95%).

## 4 Discussion

This systematic literature review and meta‐analysis quantifies the substantial mortality burden among complicated influenza patients admitted to the ICU in Europe, with results indicating that death occurs in approximately 1 out of every 4 patients. This finding is consistent with a recent Spanish surveillance system report [58], which showed an estimated ICU‐admitted mortality risk in the 2018–2019 season of 20.7%, and also an Italian surveillance report that demonstrated a mortality risk of 25.2% for patients with severe influenza cases during the same season [1].

The high heterogeneity observed in this study likely reflects variations in patient demographics, ICU practices, and healthcare policies across Europe over the decade studied, as well as differences between the selected studies (e.g., sentinel systems versus single site studies; different study periods, etc.), all of which are expected when aggregating mortality data on critically ill populations across multiple observational studies. Large differences were also seen in the sample sizes and the presence of reported risk factors (e.g., metabolic/cardiovascular/neurocognitive/other diseases, immunosuppression, obesity, etc.), though the majority of studies consisted of laboratory‐confirmed influenza. Estimates could also vary due to sources of transmission, i.e. community versus hospital‐associated acquisition, the latter of which is known to be associated with an increased risk of severe complications and death [59]. Likewise, geographic scope, including reporting capacity and the degree to which data were captured between countries [14], can make mortality rates challenging to compare, and emphasizes the need for a pan‐European study. Our use of a random‐effects model was intended to accommodate this variability, allowing us to account for differences across studies while yielding a conservative, generalized estimate that recognizes the diversity within European ICUs. The robustness of our findings is further supported by the results of the sensitivity analysis, which found similar pooled mortality risk when only studies at low risk of bias were included, indicating that the findings of the main meta‐analysis are not sensitive to risk of bias. The pooled estimate, despite heterogeneity, serves as a practical and informative measure for understanding mortality risk across diverse ICU settings, supporting health professionals and policymakers in resource planning and intervention prioritization.

This study examined several influenza seasons, including seasons that coincided with the onset of the 2009 influenza pandemic, with varying incidence and severity of disease among countries, which may have affected the mortality estimate. Most studies included the 2009 pandemic strain (A(H1N1)pdm09), which had resulted in an estimated 150,000–575,000 deaths worldwide in the first year [60]. While this pandemic outbreak resulted in an overall higher risk of adverse outcomes (i.e., ICU admission or death) compared to seasonal influenza, it has also been shown that if patients received antiviral treatment within 96 hours from symptom onset, mortality rates were lower for patients infected with the 2009 pandemic strain compared with the seasonal influenza rate for 2007 and 2008 [61]. Furthermore, reported mortality in the 2009 pandemic was higher in lower income countries and in patients with organ failure or on mechanical ventilation [62]. This suggests that resource constraints may be a key factor in mortality risk.

When considering resource constraints, increased hospitalization utilization during seasons with the influenza pandemic is a particularly significant factor affecting mortality rates [63]. For example, a US study examining the January–April 2018 influenza season reported that over half of participating ICU sites experienced critical care resource limitations [64], suggesting that many systems are not prepared for public health emergencies such as a pandemic, whether caused by influenza or another pathogen.

Pre‐existing immunity has long been demonstrated to affect influenza mortality rates: during the 2009–2010 season, hospitalization rates decreased with increasing age, even though advanced age is a known risk factor for influenza‐related hospitalization [65]. The authors concluded that in this instance, the immunity in older populations was likely due to pre‐existing immunity, which highlights the importance of vaccination in those patients at high‐risk of developing complicated influenza, especially since pre‐existing immunity to influenza has dwindled since the COVID‐19 pandemic [66].

Finally, the pandemic influenza A(H1N1)pdm09 strain displaced previously circulating seasonal H1N1 strains and has continued to circulate as the predominant H1N1 strain alongside other influenza viruses [16, 17, 18], hence the 2009–2010 season remains to be virologically relevant for the aim of this study. Additionally, the 2009 influenza pandemic significantly influenced influenza surveillance, ICU management protocols, and therapeutic strategies worldwide. Since 2009, there has been a greater focus on improving ICU care for influenza patients, which aligns well with our objective of assessing the current mortality burden and informing areas for improvement in influenza therapeutics, including antiviral treatment.

Compared to an estimated 4% mortality risk in hospital‐admitted influenza patients [67], the all‐cause ICU mortality risk of just under 25% reinforces the importance of preventing complicated influenza and therefore the continued necessity for preventative vaccinations [68, 69] and optimizing treatment. The low vaccination coverage (0%–19%) in the eight studies that reported this may have contributed to the mortality estimate seen in this meta‐analysis and further emphasizes the importance of influenza vaccination in patients at high‐risk of developing complicated influenza. Immunized patients ≥ 65 years of age have been reported to experience a 13%–35% risk reduction in all‐cause mortality, with the greatest benefit seen in elderly patients with a history of lower‐respiratory tract infection [70].

Administration of NAIs within 48 hours after the onset of symptoms of influenza is associated with decreased risk of requiring mechanical ventilation and death [71]. While there may be concerns with NAI‐resistant strains emerging, as it did for previous adamantane therapies [72], thus far NAI resistance has remained at low levels [73]. Oseltamivir resistance, conferred by a single point mutation (*H275Y*) in seasonal A(H1N1) influenza strains and reported during the 2007–2008 season [74], was a cause for concern. The emergence of influenza A(H1N1)pdm09 strain during the 2009 pandemic strain and its subsequent predominance among H1N1 strains has meant that resistance has remained low, but the threat still exists. Oseltamivir‐resistant H275Y viral infections have been shown to be susceptible to zanamivir, as zanamivir binding is unaffected by the H275Y mutation [3], underscoring the importance for a variety of available medications to treat infections such as influenza, which change and evolve rapidly, as well as the importance of rapid diagnosis and early treatment to prevent complicated influenza.

The strengths of this study included the use of a sensitivity analysis which provided similar results, confirming the robustness of the main meta‐analysis findings. The multi‐lingual eligibility criteria allowed the meta‐analysis to cover a wide range of studies. The examined studies included a high rate of laboratory‐confirmed influenza cases, providing confidence that the mortality risk reported here is due to influenza.

Among the limitations of this study is the exclusion of papers in other languages than French, German, Spanish, and English, resulting in the potential for some relevant studies to be missed. It should be noted, however, that only 17 out of the 931 screened publications were excluded for being written in a non‐included language (Figure 1). Additionally, since this study focused on European countries, the results of the meta‐analysis may be less generalizable to countries outside of Europe. The fact that the systematic literature search was conducted in PubMed only could be considered a limitation. The detailed search strategy within PubMed, however, incorporated a broad range of keywords and Medical Subject Headings terms related to influenza, ICU settings, and mortality outcomes. Additionally, manual reference checks of identified studies and relevant review articles were conducted to capture any additional pertinent studies that might not have surfaced in the initial search. Therefore, although it is possible that some studies were missed due to using a single database, we do not expect this number to be substantial and hence impact on the estimates provided. Another potential limitation may be that some studies examined small populations, which may skew mortality outcomes, and despite the best efforts to remove redundant datasets, it cannot be ruled out with absolute certainty that the included studies in this meta‐analysis did not have overlapping patient data. In addition, this study cannot fully account for any potential reporting bias for ICU‐admitted mortality in surveillance systems. Lack of information on covariates (e.g., vaccination status and treatment with NAIs) in some studies means that it is difficult to account for whether these factors may have affected the reported mortality risk, while other studies did not explicitly state the place of death, meaning that some patients may have died following discharge from the ICU.

## 5 Conclusions

This meta‐analysis quantifies the substantial mortality risk for patients admitted to the ICU and who have influenza, with death occurring in one in every four patients. This study reinforces the importance of preventing influenza through effective vaccination and treatment optimization in patients at high risk of developing complicated influenza.

## Author Contributions

The authors meet the International Committee of Medical Journal Editors' criteria for authorship, and are accountable for the integrity of the work, contributed to the writing and reviewing of the manuscript, and have given final approval for the version to be published. All authors had full access to the data in this study and are responsible for its integrity and the accuracy of the analysis. P Suárez‐Sánchez contributed to study concept/design, data acquisition, analysis, and interpretation. J Majuelos, M Hinojosa‐Campos and B Podmore contributed to data acquisition, analysis, and interpretation. I Gillespie, J Han, R Sloot and D Christensen contributed to study concept/design and interpretation.

## Ethics Statement

The authors have nothing to report.

## Patient Consent on File

This study does not include factors necessitating patient consent.

## Conflicts of Interest

Pablo Suarez‐Sanchez, Jara Majuelos, Marina Hinojosa‐Campos, Bélène Podmore are employees of OXON Epidemiology Ltd Epidemiology & Statistics, Madrid, Spain, an independent contract research organization, which received funding from GSK to design and conduct this study. Iain A Gillespie, Jennifer Han, Rosa Sloot, and Dina Christensen are employees of and may hold shares in the GSK group of companies.

### Peer Review

The peer review history for this article is available at https://www.webofscience.com/api/gateway/wos/peer‐review/10.1111/irv.70073.

## Supporting information


**Table S1:** Search strategy.
**Table S2:** Quality assessment table.
**Table S3:** Additional characteristics of selected studies.
**Table S4:** Study population characteristics.

## Data Availability

All data are presented in the manuscript and accompanying supplementary material.
