# Supplementary material for: Mortality Risk Among Patients With Influenza Illness Admitted to the ICU: A Systematic Review and Meta‐Analysis
Source: Influenza Other Respir Viruses. 2025 Mar 16;19(3):e70073. doi: 10.1111/irv.70073 (PMC11911131; doi:10.1111/irv.70073)
Supplement: Supplementary file 1 — Table S1: Search strategy. Table S2: Quality assessment table. Table S3: Additional characteristics of selected studies. Table S4: Study population characteristics. [file IRV-19-e70073-s001.docx]

# Supplementary Information

**Supplementary Table 1: Search strategy**

| **Term** | **Synonyms + related terms** | **Search strategy** | **Filter** |
| --- | --- | --- | --- |
| Event of interest | death, mortality, fatal outcome | (((death[Title/Abstract] OR fatal*[Title/Abstract] OR mortality[Title/Abstract] OR died[Title/Abstract] OR dead[Title/Abstract] OR CFR[Title/Abstract] OR Case Fatality Rate[Title/Abstract]) OR (mortality[MeSH Major Topic])) OR (fatal outcome[MeSH Major Topic])) OR (death[MeSH Major Topic]) |  |
| Condition | Influenza, flu | (Influenza[Title/Abstract] OR flu[Title/Abstract] OR ILI[Title/Abstract] OR SARI[Title/Abstract] OR H1N1[Title/Abstract] OR "A(H1)pdm09"[Title/Abstract] OR "A(H1N1)pdm09"[Title/Abstract] OR "A(H3)"[Title/Abstract] OR "A(H3N2)"[Title/Abstract] OR "A(H5N1)"[Title/Abstract] OR B/Yamagata[Title/Abstract] OR B/Victoria[Title/Abstract]) OR (Influenza, Human[MeSH Major Topic]) |  |
| Context | ICU, intensive care unit, intensive therapy unit, critical care | (((Admitted to ICU[Title/Abstract] OR ICU[Title/Abstract] OR Critical*[Title/Abstract] OR Intensive Care Unit[Title/Abstract] OR Severe*[Title/Abstract] OR Intensive Therapy Unit[Title/Abstract]) OR (Critical Care Outcomes[MeSH Major Topic])) OR (Critical Illness[MeSH Major Topic])) OR (Critical Care[MeSH Major Topic]) |  |
| Region | Europe | ((Europe[MeSH Major Topic]) OR (European Union[MeSH Major Topic])) OR (Europe, Eastern[MeSH Major Topic]) OR (Europe or European Union or EU or European Free Trade Association or EFTA or Euro*) OR (Austria or Belgium or Bulgaria or Croatia or Cyprus or Czech Republic or Denmark or Estonia or Finland or France or Germany or Greece or Hungary or Ireland or Italy or Latvia or Liechtenstein or Lithuania or Luxembourg or Malta or Netherlands or Norway or Poland or Portugal or Romania or Slovakia or Slovenia or Spain or Sweden or Switzerland or United Kingdom or UK or England or Scotland or Wales or Northern Ireland or Iceland) |  |
| Event of interest AND Condition AND Context AND Region |  |  | ("2009"[Date - Publication]: "2019"[Date - Publication]) AND (humans[Filter]) |

## Supplementary Table 2: Quality assessment table

| Bias Domain | | | | | | Total score | Risk of bias |
| --- | --- | --- | --- | --- | --- | --- | --- |
| ***Selection bias*** | | | ***Outcome*** | | |  |  |
| ***Representativeness of exposed cohort*** | ***Ascertainment of exposure*** | ***Outcome not present at study start*** | ***Assessment of outcome*** | ***Follow-up period*** | ***Adequacy of follow-up cohorts*** |  |  |
| Eligibility criteria, is the case definition adequate? |  |  | Independent/blind assessment or record linkage? | Was follow-up long enough for outcome to occur? | Are losses to follow-up related to either exposure or outcome? |  |  |
| 1. Truly representative of the average in the community, population* (i.e. surveillance systems, several influenza seasons and no specific exclusion criteria) | 1. Laboratory-confirmed influenza** | 1. Yes* | 1. Death among patients admitted to ICU is clearly defined in the ICU or hospital discharge record * | 1. Yes (until discharge from hospital) * | 1. Complete follow-up all subjects accounted for* (i.e. clearly stated and no losses in the hospitalized patients admitted to the ICU and outcome registered in all patients) |  |  |
|  | 1. Diagnosed by physician* |  |  |  |  |  |  |
| 1. Somewhat representative* |  |  |  |  |  |  |  |
| 1. Selected group of users e.g. nurses, volunteers | 1. No description | 1. No | 1. Unclear if death occurred in ICU | 1. No | 1. Subjects lost to follow-up unlikely to introduce bias* |  |  |
|  |  |  |  |  | 1. Follow up rate < 90%, no description of those lost |  |  |
| 1. No description |  |  |  |  |  |  |  |
|  |  |  |  |  | 1. No statement |  |  |
| ICU, Intensive Care Unit  Low: 3 or 4 stars in selection domain AND 2 or 3 stars in outcome domain; Moderate: 1 or 2 stars in selection domain AND 2 or 3 stars in outcome domain: High: 0 star in selection domain OR 0 or 1 stars in outcome domain  Note: A study can be awarded a maximum of one star for each numbered item within the Selection and Outcome categories. An exception is Ascertainment of the exposure which can obtain a maximum of two stars. | | | | | | | |

## Supplementary Table 3: Additional characteristics of selected studies

| **Study ID** | **Number of Seasons** | **Pandemic Season 09-10** | **Cases With ≥1 Risk Factor (%)** | **Cases With Laboratory-confirmed Influenza (%)** | **Cases With Influenza A (H1N1) (%)** | **Cases Treated With NAI (%)** | **Vaccinated Cases (%)** | **Mortality definition** |
| --- | --- | --- | --- | --- | --- | --- | --- | --- |
| **Adenji**  **2011** | 1 | Yes (only) | 63 | 100 | 19 (100) | NA | NA | ICU mortality |
| **Adlhoch 2012** | 1 | Yes (only) | NA | 100 | 100 | NA | NA | ICU mortality |
| **Akers**  **2017** | 2 | No | NA | 100 | NA | NA | NA | ICU mortality |
| **Athanasiou 2011** | 1 | No | 24 | 100 | 100 | 98 (antiviral) | NA | ICU mortality |
| **Ausselet**  **2014** | 1 | Yes (only) | 100 | 100 | 100 | NA | NA | ICU mortality |
| **Bassetti**  **2010** | 1 | Yes (only) | 100 | 100 | 100 | NA | NA | ICU mortality |
| **Bauernfeind 2013** | 4 | Yes (among others) | NA | 100 | NA | NA | NA | ICU mortality |
| **Bertolini 2011** | 1 | Yes (only) | 33 | 95 | 95 | NA | NA | ICU mortality |
| **Beumer 2018** | 1 | No | 62 | 100 | NA | 84 | NA | ICU mortality |
| **Bonmarin 2015** | 4 | Yes (among others) | 73 | 100 | 59 | NA | 17 | ICU mortality |
| **Brandsaeter 2011** | 1 | Yes (only) | NA | 100 | 100 | NA | NA | ICU mortality |
| **Brink**  **2012** | 1 | Yes (only) | 40 | 100 | 100 | 91 | NA | 28–day mortality (hospital admission) |
| **Cardeñosa**  **2011** | 1 | Yes (only) | NA | 100 | 100 | NA | NA | ICU mortality |
| **Cherifi**  **2011** | 1 | Yes (only) | 91 | 100 | 73 | NA | NA | ICU mortality |
| **Chippirraz 2011** | 1 | Yes (only) | NA | 100 | 100 | 100 | NA | ICU mortality |
| **Domínguez 2018** | 6 | No | 27 | 100 | NA | 94 | 18 | ICU mortality |
| **Drăgănescu 2019** | 1 | No | NA | 73 | NA | NA | NA | ICU mortality |
| **Gubbels 2012** | 2 | Yes (among others) | 79 | 100 | 72 | NA | 19 | 30–day mortality |
| **Heyd**  **2017** | 1 | No | NA | NA | NA | NA | NA | ICU mortality |
| **Hlavinkova 2015** | 1 | Yes (only) | NA | 100 | 100 | NA | 0 | ICU mortality |
| **Lehners 2013** | 2 | Yes (among others) | 90 | 100 | 100 | NA | NA | ICU mortality |
| **Linko**  **2011** | 1 | Yes (only) | 59 | 100 | 100 | 95 | NA | ICU mortality |
| **Lytras**  **2019** | 9 | No | NA | 100 | 59 | NA | NA | ICU mortality |
| **Martínez Ochoa**  **2010** | 1 | Yes (only) | 100 | 100 | 100 | NA | NA | ICU mortality |
| **Martin-Loeches**  **2016** | 4 | Yes (among others) | 33 | 100 | NA | NA | NA | ICU mortality |
| **Meerhoff 2015** | 3 | Yes (among others) | 66 | 100 | 66 | NA | 3 | ICU mortality |
| **Mickienė**  **2011** | 1 | Yes (only) | 33 | 100 | 100 | 100 | NA | ICU mortality |
| **Nicolay 2010** | 1 | Yes (only) | 82 | 100 | 100 | NA | NA | ICU mortality |
| **Pérez-Carrasco 2015** | 3 | No | 61 | 100 | NA | 100 | NA | ICU mortality |
| **Poeppl**  **2011** | 1 | Yes (only) | 80 | 100 | 98 | 57 | 0 | ICU mortality |
| **Rizzo**  **2016** | 5 | No | NA | 100 | 0 | NA | 16 | ICU mortality |
| **Rovina**  **2014** | 2 | Yes (among others) | 50 | 100 | 100 | 100 | 0 | ICU mortality |
| **Rowan**  **2010** | 1 | Yes (only) | 60 | 33 | 33 | NA | NA | ICU mortality |
| **Santa-Olalla Peralta 2010** | 1 | Yes (only) | 76 | 100 | 100 | 93 (antiviral) | NA | ICU mortality |
| **Scriven 2009** | 1 | Yes (only) | 100 (2 NA) | 100 | 100 | 100 | NA | ICU mortality |
| **Van Ierssel 2014** | 2 | Yes (among others) | 50 | 100 | 100 | 100 | NA | ICU mortality |
| **Viasus**  **2011** | 2 | Yes (among others) | NA | NA | NA | NA | NA | ICU mortality |
| ICU, Intensive Care Unit; NA, Not Applicable; NAI, neuraminidase inhibitor | | | | | | | | |

## Supplementary Table 4: Study population characteristics

| **Study ID** | **Study population characteristics** |
| --- | --- |
| **Adenji 2011** | Adult patients who were admitted to the hospital and who were subsequently confirmed to have contracted H1N1 |
| **Adlhoch 2012** | Patients admitted to the hospital with laboratory-confirmed pandemic influenza (influenza A[H1]pdm09) |
| **Akers 2017** | All hospitalized and consenting patients greater than 16 years of age with influenza A or B infection confirmed by polymerase chain reaction (PCR)30 from respiratory specimens (nasopharyngeal swab or broncho- alveolar lavage) in influenza seasons 2013/2014 and 2014/2015 |
| **Athanasiou 2011** | Patients, whose data were recorded, were all laboratory-confirmed influenza cases (as determined by real-time RT-PCR) who were admitted to an ICU, as well as those with a fatal outcome. |
| **Ausselet 2014** | All patients admitted to the ED were screened by a triage officer, with criteria defined as fever (> 38°C) or sensation of fever with antipyretics and at least two of the following: myalgia/headache, cough, sore throat, rhinorrhea, dyspnea (with particular emphasis on gastrointestinal and neurological symptoms). |
| **Bassetti 2010** | Patients presenting with influenza-like illness (temperature ‡38C and cough or sore throat) or other symptoms consistent with influenza, and who were hospitalized for at least 24 h between 1 July and 30 November 2009 |
| **Bauernfeind 2013** | All patients admitted for signs, symptoms, or complications due to influenza and a respiratory sample positive for influenza virus by polymerase chain reaction (PCR) |
| **Bertolini 2011** | Patients with a suspected or proven A(H1N1) infection |
| **Beumer 2018** | Patients who were admitted to the hospital with clinical symptoms due to an acute infection with Influenza A or B |
| **Bonmarin 2015** | Laboratory-confirmed influenza cases admitted to ICU in mainland France that were reported during the pandemic (2009/10) and the subsequent three winter seasons. |
| **Brandsaeter 2011** | All hospitalised patients above 18 years of age with suspected H1N1 Influenza A were enrolled in the study |
| **Brink 2012** | All individuals with laboratory-confirmed infection with novel influenza A (H1N1) virus in Sweden (population 9.4 million) admitted to a Swedish ICU. |
| **Cardeñosa 2011** | All confirmed hospitalized cases of pandemic influenza which presented criteria of severe disease. |
| **Cherifi 2011** | All adult patients hospitalised due to microbiologically confirmed influenza A. |
| **Chippirraz 2011** | Patients older than 18 years, with influenza A confirmed by PCR of nasopharyngeal swab |
| **Domínguez 2018** | Reported cases of laboratory-confirmed severe hospitalised influenza in persons aged ⩾18 years during six influenza seasons (2010–2011 to 2015–2016) |
| **Drăgănescu 2019** | Patients admitted for acute ILI were included if they were resident in the Bucharest-Ilfov region, had been hospitalized for at least 24 h, and had onset of symptoms within 7 days before admission. |
| **Gubbels 2012** | Patients in a Danish ICU with laboratory-confirmed influenza A(H1N1)pdm09 or with ILI after close contact with a person with laboratory-confirmed influenza A(H1N1)pdm09. |
| **Heyd 2017** | Patients with influenza-like illness (ILI) in combination with laboratory-confirmed influenza were included |
| **Hlavinkova 2015** | Adults and children with clinical symptoms of influenza with laboratory-confirmed pandemic influenza virus A(H1N1)pdm09 |
| **Lehners 2013** | All patients admitted to the hospital, with laboratory-confirmed influenza A(H1N1) pdm09 infection |
| **Linko 2011** | All patients admitted to Finnish ICUs with high suspicion of or confirmed pandemic Influenza A(H1N1) infection during the Finnish H1N1 outbreak between 11 October 2009 and 31 December 2009. High suspicion of Influenza A(H1N1) was defined as typical clinical picture suitable for H1N1 infection leading to administration of oseltamivir treatment. The diagnosis was confirmed by real-time reverse transcription polymerase chain reaction (PCR) test |
| **Lytras 2019** | All cases of severe influenza at the country-level (defined as a laboratory confirmed case where the patient has been either (1) hospitalized in an Intensive Care Unit (ICU), (2) mechanically ventilated in a regular hospital ward or (3) has died in hospital). |
| **Martínez Ochoa 2010** | All cases with 2009 H1N1 influenza in La Rioja during study period. |
| **Martin-Loeches 2016** | All patients admitted to the ICU with influenza-like symptoms. Patients under the age of 16 years and patients admitted from nursing homes or other healthcare facilities were excluded. |
| **Meerhoff 2015** | Hospitalised patients meeting a syndromic SARI case definition |
| **Mickienė 2011** | Adult (≥18 years of age) cases of the pandemic 2009 influenza A (H1N1) virus admitted to three hospitals located in Kaunas |
| **Nicolay 2010** | Adult patients admitted to an Irish ICU with confirmed or probable pandemic (H1N1) 2009 infection |
| **Pérez-Carrasco 2015** | SARI patients admitted to the ICU. A SARI case was defined as sudden onset of fever (>38 ◦C), cough or sore throat in the absence of any other diagnosis and shortness of breath or difficulty in breathing |
| **Poeppl 2011** | In- and outpatients with pandemic H1N1 influenza in Austria. Only patients with H1N1 infection confirmed by real time reverse transcriptase polymerase chain reaction (RT-PCR) assay or by Quick test were included in the analysis. |
| **Rizzo 2016** | Patients hospitalised with laboratory-confirmed influenza B virus |
| **Rovina 2014** | Subjects who presented to the emergency department with an influenza-like illness and were investigated for the 2009 influenza A (H1N1) virus and who were then admitted to the RIU due to underlying chronic conditions or to treat emerging complications. |
| **Rowan 2010** | All patients (adult or paediatric) were included in SwiFT if they were either confirmed or suspected pandemic H1N1 patients referred and assessed as requiring critical care; or non-H1N1 patients referred and assessed as requiring critical care (under usual/non-pandemic circumstances), but not admitted to a critical care unit in the hospital where referred and assessed. |
| **Santa-Olalla Peralta 2010** | Cases of pandemic (H1N1) 2009 virus infection hospitalised in intensive care units (ICUs) notified to CCAEs |
| **Scriven 2009** | Adults with confirmed swine flu by polymerase chain reaction (PCR) |
| **Van Ierssel 2014** | Patients admitted at the ICU with severe influenza A(H1N1)2009_Table provided information for adults only |
| **Viasus 2011** | Hospitalized adults with confirmed influenza A (H1N1) |
